# Draft Genome Sequences of *Pseudomonas* sp. Strains Isolated from Wheat in Germany

**DOI:** 10.1128/MRA.00178-19

**Published:** 2019-05-09

**Authors:** Jürgen Hollmann, Erik Brinks, Christine Schwake-Anduschus, Gyu-Sung Cho, Charles M. A. P. Franz

**Affiliations:** a Department of Safety and Quality of Cereals, Max Rubner-Institut, Federal Research Institute of Nutrition and Food, Detmold, Germany; b Department of Microbiology and Biotechnology, Max Rubner-Institut, Federal Research Institute of Nutrition and Food, Kiel, Germany; University of Arizona

## Abstract

Three chloramphenicol-resistant *Pseudomonas* sp. strains were isolated from wheat grain in Germany on rose Bengal agar. The draft genome sizes ranged from 5,924,931 to 6,124,470 bp. All the isolates possessed genes for efflux pumps that might be responsible for an intrinsic chloramphenicol resistance. No acquired antibiotic resistance genes in these strains could be determined.

## Abstract (precis)

Three chloramphenicol-resistant *Pseudomonas* sp. strains were isolated from wheat grain in Germany on rose Bengal agar. The draft genome sizes ranged from 5,924,931 to 6,124,470 bp.

## ANNOUNCEMENT

Species belonging to the genus *Pseudomonas* (*sensu stricto*) are members of the gammaproteobacteria and are Gram-negative catalase-positive rods (1). These bacteria exhibit remarkable metabolic and physiologic versatility, which enables them to effectively colonize a wide range of terrestrial and aquatic habitats (2, 3). In addition, pseudomonads are important, as they are pathogenic toward plants and humans. Apart from some species being important plant pathogens, isolates from other species (e.g., P. aeruginosa, P. fluorescens, P. putida, and P. stutzeri) interact with plants and can contribute to plant health by antagonizing plant-pathogenic microorganisms, thereby directly influencing plant disease resistance and promoting growth (2, 4). In regular bacteriological screens of whole-grain wheat samples after harvest, rose Bengal agar medium (containing 100 µg/ml chloramphenicol as the antibacterial agent) was used for the selective enumeration of yeasts and fungi. The appearance of some chloramphenicol-resistant bacterial colonies from 1 g of wheat grain sample was, however, noticed after 5 days of incubation at 25°C. Three strains isolated from this medium in 2016 were investigated using whole-genome sequencing in order to identify the strains and the chloramphenicol-resistance gene(s).

For whole-genome sequencing, single colonies were cultured in Luria-Bertani broth overnight at 30°C. The total genomic DNA of the *Pseudomonas* sp. strains was extracted using the peqGOLD bacterial DNA kit (Peqlab, Erlangen, Germany) according to the manufacturer’s instructions. The sequencing library was prepared with an Illumina TruSeq Nano DNA prep kit (Illumina, San Diego, CA, USA) and run on an Illumina MiSeq instrument with 2 × 251-bp paired ends. A total of 1,750,480 paired ends and 52,100 single-end sequence reads were obtained from three samples with coverage that ranged from 23- to 38-fold. The low-quality reads and adapter sequences were removed with Trimmomatic version 0.36 (5). The reads were *de novo* assembled using SPAdes version 3.13.0 (6) with the parameters k-mer 77 and careful and a minimum contig length of 500 bp. The draft genome sequences were annotated using the NCBI Prokaryotic Genome Annotation Pipeline (7) and analyzed for gene features with PATRIC (8). Except for the SPAdes pipeline, default parameters were used for all other software. The genome features and the quality information of the *de novo* assembly are described in Table 1. The genomes consisted of 80 to 153 contigs per strain, and the *N*_50_ values ranged from 75,064 to 240,160 bp (Table 1). To identify these strains, the complete 16S rRNA gene sequences were extracted from the PATRIC data set and applied in the EzTaxon pipeline (9). The 16NI and 133NRW strains were identified as P. orientalis with 99.86 and 99.73% 16S rRNA gene similarities, while strain 770NI showed 100% nucleotide identity with the *Pseudomonas* sp. strain CP019856_s 16S rRNA gene. Using the ResFinder server version 3.1 (10), none of the isolates were found to carry any acquired chloramphenicol resistance genes, such as genes involved in chloramphenicol acetylation (*cat* genes). All three isolates, however, contained genes encoding a MexAB-OprM and a MexEF-OprN efflux pump; these were previously described in connection with intrinsic chloramphenicol resistance in P. aeruginosa strains. In addition, the MexEF-OprN efflux pump genes were found to be inducible by chloramphenicol (11). The presence of these genes may explain the chloramphenicol resistance of the isolates investigated.

**TABLE 1:** *De novo* assembly of three *Pseudomonas* strains isolated from wheat grain

Strain	GenBank accesion no.	SRA accession no.	No. of contigs	No. of CDS^*a*^	Genome size (bp)	*N*_50_ (bp)	GC content (%)
16NI	SGFD00000000	SRR8607514	123	5,413	5,972,457	121,728	60.65
133NRW	SGFE00000000	SRR8607513	153	5,647	6,124,470	75,064	60.48
770NI	SGFF00000000	SRR8617823	80	5,485	5,924,931	240,160	60.17

a CDS, coding DNA sequences.

### Data availability.

The whole-genome sequences of P. orientalis strains 16NI and 133NRW and *Pseudomonas* sp. strain 770NI were deposited in DDBJ/ENA/GenBank under the accession no. SGFD00000000, SGFE00000000, and SGFF00000000, respectively. The raw reads can be found in the SRA with no. SRR8607514 (16NI), SRR8607513 (133NRW), and SRR8617823 (770NI).
